# Hardware Mechanism for Energy Saving in WiFi Access Points

**DOI:** 10.3390/s19214745

**Published:** 2019-11-01

**Authors:** Juan Pablo García Baquerizo, Alvaro Suárez, Elsa Macias, Edgar Salas

**Affiliations:** 1 University of Las Palmas de Gran Canaria, Architecture and Concurrence Research Group, Institute of Sciences and Cybernetic Technologies, 35011 Canary Islands, Spain; juan.garcia162@alu.ulpgc.es (J.P.G.B.); elsa.macias@ulpgc.es (E.M.); 2 Computer Engineering Management Department, Santamaría Campus Guayaquil University, Guayaquil 090150, Ecuador; 3 Faculty of Communication, Espíritu Santo University, Guayaquil 090150, Ecuador; esalas@uees.edu.ec

**Keywords:** wireless fidelity, access point, energy saving, power router, battery, power consumption optimization

## Abstract

Wireless fidelity (WiFi) networks are deployed in several varied environments all around the World. Usually, the wireless fidelity access points are always on in houses and other small companies. In buildings of large companies and public organizations and in university campuses the number of access points is elevated; they are powered using power over the ethernet and are always on. Consequently, they consume a considerable amount of electric energy. The last versions of the International Electric and Electronic Engineers 802.11 standardized procedures to save energy in a wireless fidelity terminal but not in the access point. We designed a formal method to show when energy can be saved in wireless fidelity access points considering different power supplies for the access point: an electric energy battery and a standard voltage supply. We use an external battery that stores electric energy during an interval of time from a standard voltage supply (Charge period). After that interval (Discharge period), the energy supply for the access point is the external battery. Those intervals of time are repeated sequentially (Charge and Discharge cycles). We verified our formal model implementing a hardware circuit that controls the power supply for the access point. The amount of energy saving for a large number of of access points during a long period of time is considerably high.

## 1. Introduction

Wireless fidelity (WiFi) networks [1] are massively used today in smart homes [2,3], enterprises, public buildings, university campuses, and smart cities [4,5]. In those domains, usually WiFi access points (AP) are always on, consuming a large amount of energy.

The Institute of Electrical and Electronics Engineers (IEEE) 802.11ac WiFi standard [6] defines a power saving mode (PSM) for the WiFi terminal (WT) during an idle time (defined by the user), but it does not define any rule for energy saving in the AP. In the smart city domain, changes to IEEE 802.11e (included in IEEE 802.11ac) [7] have been proposed to reduce power consumption of WTs when communicating sensor data in the Internet of Things (IoT) [8,9] where, as mentioned in [10], WiFi is considered as the bottleneck to reducing the power consumption of an IoT device. Device-to-device (*D2D*) communications allow the minimization of the amount of time the WiFi interface of the WTs are active communicating data. In [11] the programming of beacon intervals delivery is analyzed for efficiently reducing the energy consumption of IoT devices using WiFi direct. In [12] several recommendations for energy saving are presented. An energy-saving mechanism taking parts of those recommendations are proposed in [13] for machine-to-machine (M2M [14]) communications taking the advantage of idle cycling technique. In mobile cloud computing for IoT, a cloudlet is proposed for processing data close to the WT using WiFi instead of long-term evolution to communicate data [15] for energy saving. We are interested in an energy-saving mechanism that can be generally applied instead of customized to specific domains.

Planned for this year, IEEE 802.11ax (WiFi 6) will provide 6 to 11 Gbps, which implies APs that will consume more energy than actual APs. For that reason, it allows the WT to negotiate the target wake up time (TWT) [16] to reduce the energy consumption [17] of WT but it does not specify anything for energy consumption in APs.

Some information and communication technology (ICT) companies [18] define energy-efficient powered device APs receiving energy from power sourcing equipment (PSE) like HPE 2520G-24-PoE and HPE 2920-48G-PoE+ [19] using power over ethernet (PoE) or directly from the electrical network. Those PSEs provide power between 15.4 W and 90 W for ethernet wires of 100 m considering standards such as IEEE 802.3af, 802.3at y 802.3bt [20]. Those initiatives do not avoid that the energy consumption of WiFi AP is between 2% and 10% of world power consumption [21] at present and could be 1700 TWh at the end of 2030 [22] (ICT is responsible for 2% to 2.5% of carbon monoxide (CO) emissions in the world [23]). We focus on a hardware mechanism for energy saving in WiFi APs independent of the standard version and kind of AP.

### 1.1. Related Work: Energy Saving in Wireless Fidelity (WiFi) Access Points (APs)

Energy consumption of WiFi APs depends on several parameters [24]: its electric power consumption, number of connected and transmitting WTs, and range of coverage. Several techniques have been used to reduce the energy consumption.

The reduction of idle pending combining the monitoring of WiFi downlink and low-power wake-up radio is simulated in [25] achieving considerable reduction of energy consumption. We implemented a hardware mechanism but not considering those techniques. WiFi energy profiles in WT acting as AP (tethering) are used to pass the WiFi interface to sleeping state for 90% of communication time without affecting performance [26]. We do not consider tethering; we apply our mechanism to any WiFi AP that could have an ethernet link to Internet.

Energy harvesting [27] and time division multiplexing [28] techniques in mobile networks optimize energy saving considering the time of communication and the time needed to gain the access to the mobile channels (allocated in different frequencies) detecting inactive mobile terminals. We take advantage of inactive WiFi APs to shorten the battery charging time.

Deactivation of the WiFi interface of the AP when a long period of inactivity in the network is detected is another technique. In [29] associations of WT are monitored in order for the AP to estimate the probability of new associations of WT in a short period of time. When that estimation detects no associations will be produced in the short-term future, the WiFi interface of the AP are deactivated for a short period of time. For dense WiFi-controlled networks, in [30] two algorithms are proposed to deactivate the WiFi interfaces of a master AP when a successful reassociation of WTs to slaves AP could be achieved during a prefixed amount of time. A communication delay could be achieved with that technique. We do not deactivate the WiFi interface of the AP in order to not delay communications.

The real time kernel Energy Neutral Operating system (*EnOS*) [31] adapts energy consumption of high-end APs triggering the energy supply from an additional battery. Our hardware mechanism does not need EnOS or other software and can be applied to any AP.

Our hardware mechanism can be applied to APs used in smart city as part of a wireless mesh network (WMN) based on IEEE802.11s. Some research works suppose the APs in the WMN are an electrical network or electrically powered by a PSE with PoE. In [32] are analyzed some routing characteristics the WTs must preserve in WMN to maintain routing paths when they will be in sleep state (they must support efficiently WiFi beacon communication and synchronization). In [33] the strength pareto evolutionary algorithm for routing is designed to optimize the energy consumption of APs in the WMN. In [34] the influence of idle pending of PSM is analyzed to prevent degradation of quality of service (QoS) and quality of experience (QoE). The work in [35] implemented experimental tests of energy consumption in a WMN with few APs and simulated the behavior for a high number of APs connected to a solar panel. Our mechanism is clear to the kind of electrical supply and it is applied when the WiFi AP will be not communicating user data.

Our mechanism can be used in applications of smart home like those [36,37] that controls turning on and off electrical bulbs on a building depending on the amount of people in a room using infrastructure WiFi APs information of WT in the building. Applying our mechanism, energy is mainly saved when no people are in the room.

### 1.2. Main Contribution

The main idea of our mechanism is to show that it is possible to save energy in any kind of WiFi AP without using extra software and using a standard battery and an extra electronic circuit that control the energy supply for the AP: the battery or any other standard voltage supply (electrical, PSE or solar panel). The battery and electronic control circuit are externally docked to any WiFi AP. Our mechanism continuously tries to shorten the charge period of the battery and to enlarge the discharge period of the battery. When the battery is being charged, the AP will use the standard electrical supply. After that period of time the battery will be discharged supplying electric energy for the AP. That cycle is continuously chained (change and discharge cycles).

The main contributions of our paper are:

- A formal model that shows that if the discharge period is larger than the charge period then our Mechanism will achieve energy saving. That parametric model is useful to adapt the implementation of our Mechanism to any WiFi AP taking into account its particular conditions.
- The implementation of our electronic control circuit for controlling the supply of a WiFi AP from a PSE supply or our external battery. The implementation of the control circuit is guided by our formal model. We present a general schematic of an easy to implement electronic circuit based on ordinary components. The need of that hardware is to control the charge and discharge periods in a simple way complementing other existing software or firmware techniques.
- The realization of a number of experimental tests for verifying the formal model and the implementation of our hardware mechanism. Those experimental tests reveal that it is possible to save energy with our mechanism. The projection of results for a number of hours shows that a considerable amount of energy could be saved.

The structure of the rest of the paper is the following: Section 2 presents the main idea of our novel mechanism, a formal model showing when energy saving could be possible and the hardware implementation of the Mechanism. In Section 3 we present the experimental tests of our mechanism. Section 4 discusses how experimental results verify our formal model and project results for energy saving in a greater amount of time and WiFi APs. Finally, we sum up some conclusions.

## 2. The Mechanism for Energy Saving in WiFi AP

**Figure 1:**
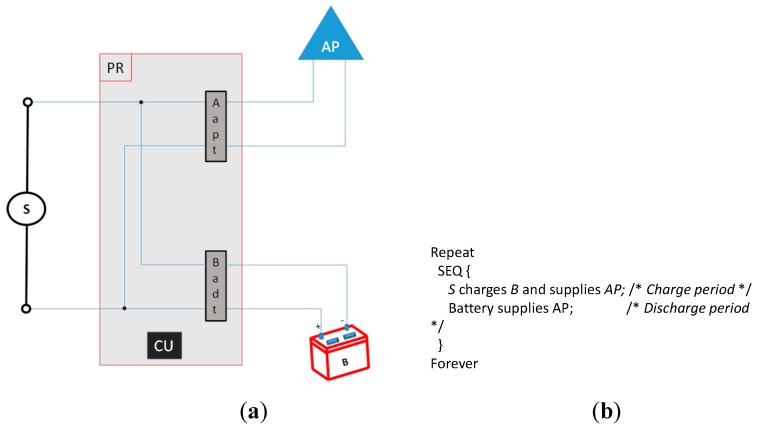
(**a**) Graphic schema of the wireless fidelity access point (WiFi AP) and main components of our novel mechanism for energy saving. (**b**) Processing model: chained alternating charge and discharge periods.

Figure 1a shows a graphic schema of the components of our mechanism applied to a generic WiFi AP: The voltage supply (S) attacks the power router (PR) which controls the voltage supply of the AP using a control unit (CU) (it basically switches S and the Battery (B) supply for the AP), and adapts the voltage and current for the AP (Aadt) and for B (Badt). Figure 1b shows the processing model algorithm: in the charge period, S charges B and supplies AP, in the discharge period, B supplies AP. The CU properly controls the Aadt and Badt components in both periods.

### 2.1. Mathematical Model Analysis

We modelled the theoretical conditions that must be accomplished to obtain energy saving comparing the power consumption of the system in Figure 1 between using our mechanism and not using our mechanism. We considered the transition between charge and discharge (and vice versa) periods takes a negligible amount of power consumption and for that reason we do not model it. That transition also does not affect the performance of AP transmitting WiFi beacons.

**Figure 2:**
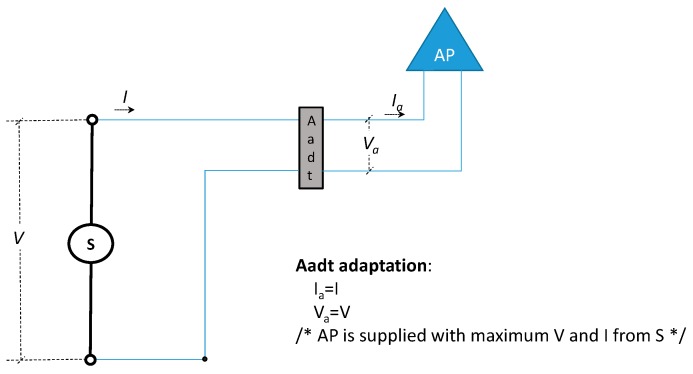
Graphic and formal model of our system without our mechanism.

Where *T* > 0 is an amount of time during the test of energy saving, *V* is the constant and continuous voltage supplied by *S* and *I* the corresponding current. Figure 2 shows our system without our mechanism and the function of *Aadt* component (we include it only for uniformity in the discussion). During *T* the power consumption (*P*) of the AP is:

(1) $$P=\int_{0}^{T}V_{a}\left(t\right)\;I_{a}\left(t\right)\;dt=\int_{0}^{T}V\left(t\right)\;I\left(t\right)\;dt$$

**Figure 3:**
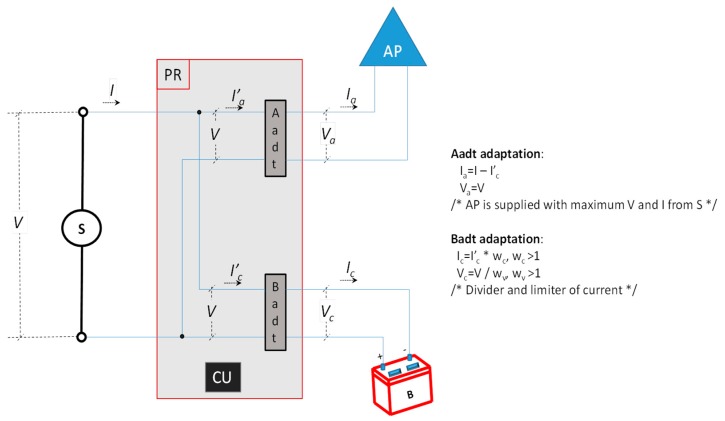
Graphic and formal model of our system with our mechanism working in the charge period.

For analysing the power consumption (*P’*) with our mechanism we differentiate the charge and discharge periods. Figure 3 shows the name of voltages and currents and the adaptation of them realized in Aadt and Badt components in the Charge period. Where, *w_v_* is a charger voltage divider factor (Badt), which depends on the internal components and the voltage value required to charge the battery, and *w_c_* is a current divider factor of the charger (Badt), which depends on the internal components and the value of the current needed to charge the battery.

If the Charge period lasts *T_c_* units of time, then the power consumption in the AP will be: $\int_{0}^{T_{c}}V_{a}\left(t\right)\;I_{a}\left(t\right)\;dt$ and the power consumed by *B* will be: $\int_{0}^{T_{c}}V_{c}\left(t\right)\;I_{c}\left(t\right)\;dt$. Adding those powers, considering the adaptation of Aadt and Badt and ignoring the power consumed in the *PR* (we consider it is negligible), and supposing the *V* is enough to supply the AP and charge the B, we will obtain:

(2) $$\begin{array}{ll} P^{\prime } & =\int_{0}^{T_{c}}V_{a}\left(t\right)\;I_{a}\left(t\right)\;dt+\;\int_{0}^{T_{c}}V_{c}\left(t\right)\;I_{c}\left(t\right)\;dt\\ & =\int_{0}^{T_{c}}V\left(t\right)\;\left(I\left(t\right)-\frac{I_{c}\left(t\right)}{w_{c}}\right)\;dt+\;\int_{0}^{T_{c}}\frac{V\left(t\right)}{w_{v}}\;I_{c}\left(t\right)\;dt\\ & =\;\int_{0}^{T_{c}}V\left(t\right)\;I\left(t\right)\;dt-\frac{1}{w_{c}}\;\int_{0}^{T_{c}}V\left(t\right)\;I_{c}\left(t\right)\;dt+\frac{1}{w_{v}}\;\int_{0}^{T_{c}}V\left(t\right)\;I_{c}\left(t\right)\;dt\\ & =\;\int_{0}^{T_{c}}V\left(t\right)\;I\left(t\right)\;dt+\left(\frac{1}{w_{v}}-\frac{1}{w_{c}}\right)\;\int_{0}^{T_{c}}V\left(t\right)\;I_{c}\left(t\right)\;dt \end{array}$$

**Figure 4:**
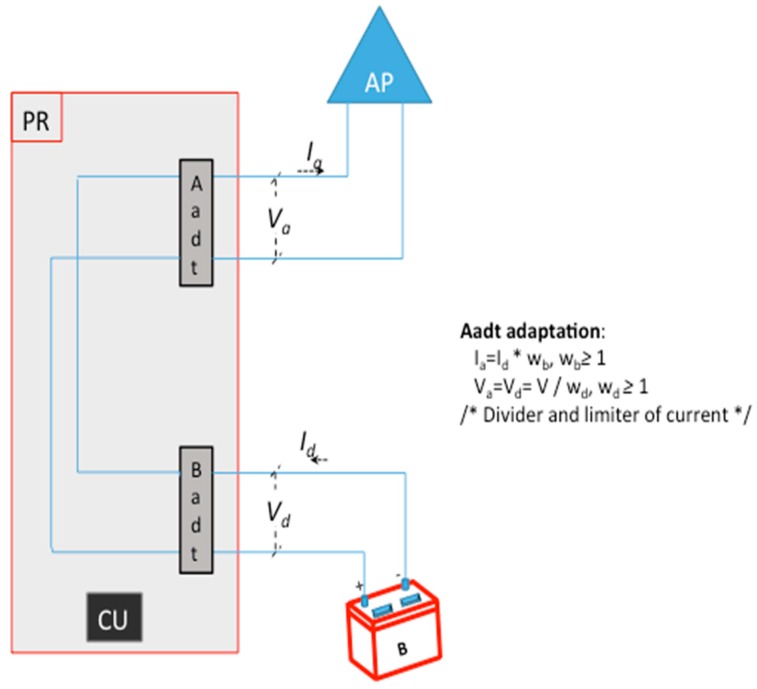
Graphic and formal model of our system with our mechanism working in the discharge period.

Figure 4 shows the name of voltages and currents and the adaptation of them realized in Aadt (Badt does not transform its inputs) component in the discharge period. Where *w_d_* is a voltage divider with reference to the Aadt and *w_b_* is a multiplication factor with reference to the Aadt.

If the discharge period lasts *T_d_*, then the power consumption in the AP will be:

(3) $$P^{\prime \prime }=\int_{0}^{T_{d}}V_{a}\left(t\right)\;I_{a}\left(t\right)\;dt=\int_{0}^{T_{d}}\frac{V\left(t\right)}{w_{d}}\;\;I_{d}\left(t\right)\;w_{b}dt=\;\frac{w_{b}}{w_{d}}\int_{0}^{T_{d}}V\left(t\right)\;I_{d}\left(t\right)\;dt$$

• Optimizing *T_c_* and *T_d_*: Let us now consider *T = T_c_ + T_d_*, that is, we only consider one charge and one discharge period for optimizing *T_c_* and *T_d_* in order to obtain the energy saving. We argue that, in general, energy saving needs that *T_c_* be minimized and *T_d_* be maximized obtaining the appropriated values of coefficients named with letter *w* above. The *T_c_* can be minimized optimizing $w_{v},$ $w_{c}$ and maximizing $I_{c}$ in *P’* maintaining the AP working correctly. The *T_d_* can be maximized optimizing $w_{d},$ $w_{b}$ and minimizing $I_{d}$ such as the AP that could work with a minimum of capability. The best values of coefficients *w* can be obtained in practice calibrating the work of the AP. The formal expressions of $I_{c}$ and $I_{d}$ are well known. So, next we focus on how to optimize their values.

(4) $$I_{c}\left(t\right)=\{\begin{array}{cc} I_{e}\left(t\right)=\overset{n}{\underset{i=0}{\sum }}w_{i}\;t^{i}; & 0<t\leq T_{e}\\ w_{t}; & T_{e}<t\leq T_{ct}\\ I_{p}\left(t\right)=w_{o}\;t^{-w_{r}}\;\left(w_{o}>0\;and\;w_{r}>0\right); & T_{ct}<t\leq T_{c} \end{array}$$

The values of *w_i_*, *w_t_*, *w_o_*, *w_r_* and temporal limits: *T_e_* y *T_ct_* determine the minimum value of *T_c_*. Those parameters depend on the battery fabric. In general, the safety conditions must be taken into account to adjust their values.

(5) $$I_{d}\left(t\right)=w_{s}\;t+w_{f}$$

where $w_{s}\approx 0$ y $w_{f}>0$. Which means that its slope ($+w_{s})$ is positive and very little. This allows the battery to start working with a high current value ($w_{f}>0$) and to maintain working the AP in correct and safe margins. An initial voltage must be supplied for that initial current value be assured.

• Optimizing energy saving in several charge and discharge cycles: let us now consider *k* (0≤k≤K) charge and discharge consecutive cycles. That is, *T* = *k* (*T_c_* + *T_d_*). The main idea is to observe which conditions must be accomplished for energy saving (minimize the amount of power *S* supplies). Let us work with mean values of *P*, *P*′ and *P*″:

(6) $$\bar{P}=\bar{V}\;\bar{I}\;T$$

(7) $$\bar{P^{\prime }}=\bar{V}\;\bar{I}\;T_{c}+\left(\frac{1}{w_{v}}-\frac{1}{w_{c}}\right)\bar{V}\;{\bar{I}}_{c}T_{c}$$

(8) $$\bar{P^{\prime \prime }}=\frac{w_{b}}{w_{d}}\bar{V}\;{\bar{I}}_{d}T_{d}$$

*WoS_k_* is the difference of power consumption with and without our mechanism in charge and discharge cycle *k* (${\langle \bar{P}-\bar{P^{\prime }}\rangle }_{k}$), and **WoS** is a vector which entries are positive, negative or zero values depending on where a save, waste or null energy saving was obtained in each iteration k. The sum of all values of **WoS** results in the save or waste of energy in *k* charge and discharge cycles. If it is assured that in each iteration we obtain energy saving, then, on average we trivially will obtain energy saving in the charge and discharge cycle *k* (more restrictive case). Let us now show the conditions under the energy saving is obtained in that case:

(9) $$\bar{V}\;\bar{I}\;T-\;\bar{V}\;\bar{I}\;T_{c}-\left(\frac{1}{w_{v}}-\frac{1}{w_{c}}\right)\bar{V}\;{\bar{I}}_{c}T_{c}>0$$

That is,

(10) $$\bar{I}\;T_{d}>\left(\frac{1}{w_{v}}-\frac{\;1}{w_{c}}\right){\bar{I}}_{c}T_{c}$$

As ${\bar{I}}_{c}<\bar{I},$ the condition $T_{d}>T_{c}$ will be achieved when $w_{v}>w_{c}.$ It is shown that, theoretically we can calculate the values of coefficients *w* that will result in energy saving after *k* charge and discharge cycles. Finding an optimal analytical solution to the above equations is very difficult. Simulating the equations using probability distribution for entries of WoS could illustrate more about the dimension of values for coefficients *w*. We focus in the demonstration that it is possible to calibrate the values of coefficients *w* in a practical implementation for obtaining energy saving.

### 2.2. Hardware Implementation of the Mechanism

**Figure 5:**
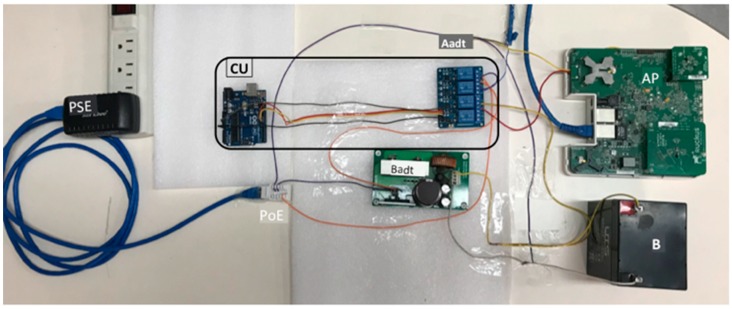
Components of the system that implemented our hardware mechanism.

Figure 5 shows a photo of the hardware implementation of our mechanism. The *S* used was a PSE that supplied 46.7 V and 410 mA (*I*) from a RJ45 Ethernet connector with PoE. The *I* attacked the AP (*I’_a_* transformed by Aadt component) and the battery (*I’_c_* transformed by Badt component).

The AP used was a standard WiFi Ruckus AP Zone Flex 7363 smart IEEE 802.11n. It is provided with an adaptive antenna technology, PoE Class 0 IEEE 802.3af standard, dual band (5 Ghz and 2.4 Ghz) and maximum electric power of 12.95 W. PoE power uses terminals 4, 5, 7 and 8 of the RJ45 connector. Terminal 7 was connected to the Badt component and terminal 8 was connected to Aadt component. Terminal 4 was connected to the CU.

The battery selection was not easy because, to our best knowledge, there is not a standard approach to finding the customized battery for different APs, from different manufacturers. We did several tests until it was found the best option for finding the best values for coefficients of Aadt and Badt components. We first chose a battery of 3.6 V and 800 mAh but it could not maintain Ruckus AP working. Then we tested a battery of 4 V and 2400 mAh; the Ruckus AP could achieve ignition and work during 8 m, which was clearly not enough. Finally, we tested a 12 V and 5000 mAh sealed lead acid battery that was able to maintain the Ruckus AP working for long periods of time. For designing the Badt component in each case we used direct current (DC)-DC standard converters (integrated circuit (IC)) avoiding its overheating with adequate thermal proteccions (possible overheating that occurs during the battery charging cycles).

In order to implement the PR, we also used standard ICs: (a) CU: we used an Arduino one R3 microcontroller board based on the ATmega328P [38] and a 4 channel relay module. (b) Aadt: its functionality is to limit the current that feeds the AP if necessary, depending on the model of AP used in the WiFi network. (c) Badt: It was used the LM 2576 IC [39] that is a voltage regulator, with capacity to control loads of up to 3 A. Its input oscillates between 40 and 60 V (DC) and the possible outputs are 3.3, 5, 12, and 15 V with an adjustable output version. It tolerated ±4% on the output voltage and ±10% on the oscillator frequency and allowed thermal shutdown for total protection in fault conditions. The outlet of the Badt generated a regulated voltage of 14.27 V for charging the battery during *T_c_*.

## 3. Experimental Results: Testing our Mechanism

**Figure 6:**
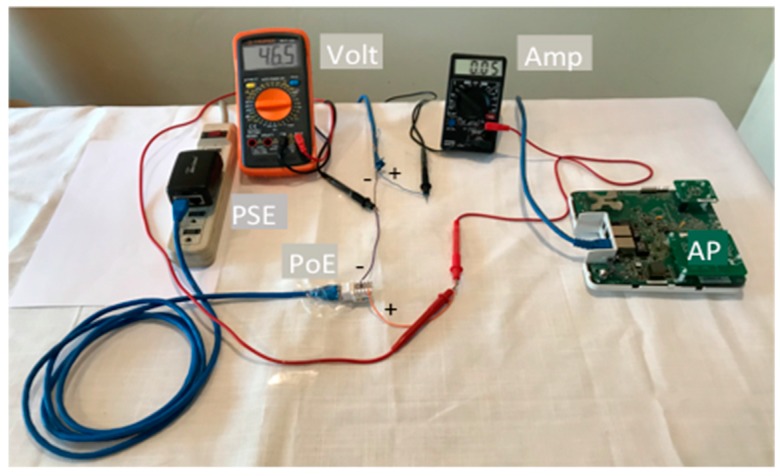
Measuring current and voltage supply to the AP alone and only transmitting beacons.

The calibration of the parameters (*w*) identified in Section 2 took us around 2 months (4 h per day approximately) because it was a complex process. About 20 tests were executed, with different types of batteries and voltage adapters. In the first test we searched for safety values of current and voltage supply for the *Ruckus AP* which only transmitted signaling WiFi beacons (no user data). We found 46.2 ≤ *V* ≤ 46.7 V and 0.03 ≤ *I* ≤ 0.41 A. Figure 6 shows the voltmeter (volt), and amperemeter (amp) measurements (46.5 V and 0.05 A). Those measurements were the ones with which the Ruckus AP was able to work. The measurements were done connecting tips of red cables of the volt and the amp to the positive (+) orange cable of PoE. The tip of black cable of the Volt was connected in the joint of negative poles of PoE (lilac cable) and Ruckus AP negative RJ45 cable (brown). The tip of a black cable of amp was connected to a positive cable of RJ45 of the Ruckus AP (blue).

**Figure 7:**
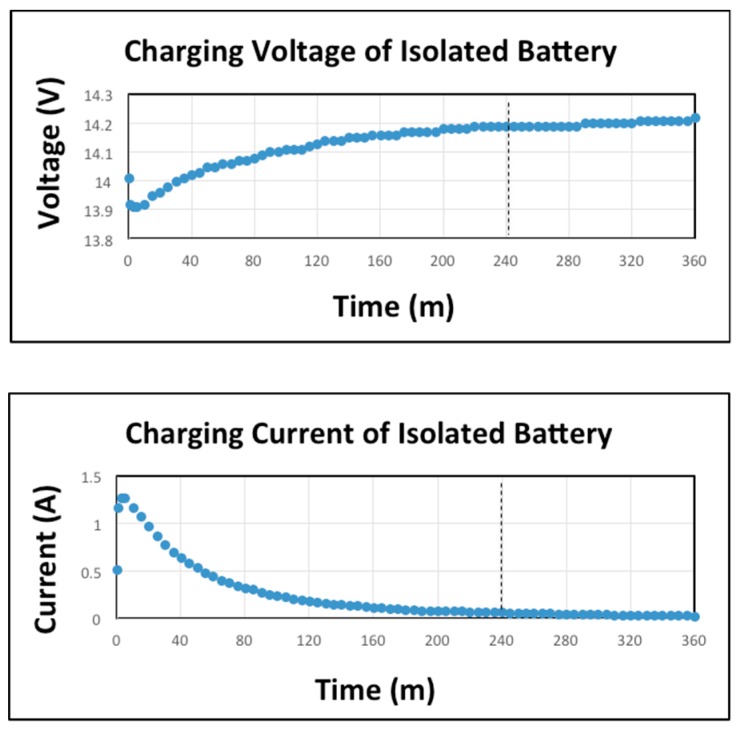
Charging current and voltage of isolated battery.

Find out the high and minimum working values of *V* and *I* for the Ruckus AP, we tested the time evolution of the charging current and voltage of the chosen battery in standalone. We did this to confirm the theoretical values found in the literature, to observe correct working of the charging process and to observe particular details of that battery. Initially the battery had a vacuum voltage (before starting the charging) of 12.15 V (Figure 7). After a rapid growth (slope of 0.253), at 3 m, the *I* reached the maximum value of 1.27 A. That maximum value was maintained for a very short period of time. Then a prolonged fall was produced until 240 m when *I* reached 0.02 A. In that period of time *V* reached 14.19 V. We considered that in 240 m the battery was charged, but we continued measuring the *V* and *I* until 360 m. In that period the *I* maintained constant and *V* increased 0.03 V.

Determining *V* and *I* for the *Ruckus AP* only transmitting WiFi beacons and the characteristics of *T_c_* for the battery in standalone, we designed and tested two different kind of experiments for verifying our formal model and that energy saving was possible with our mechanism:

- *Analysis of the T_c_ using our mechanism*: the objective was to analyze the behavior of the battery charging current and voltage when the supply *S* energized the *Ruckus AP* and the battery simultaneously. We observed the behavior of *I_c_, V_c,_ I_a_* and *V_a_*.
- *Analysis of the T_d_ using our mechanism*: the objective was to analyze if the *Ruckus AP* could work being energized by *B* and to determine *T_d_*. We observed the behavior of *I_d_* and V*_d_*.

The experiments were conducted maintaining the Ruckus AP transmitting only beacons. The shape of the current and voltage curves in the experiments behaved similarly. The differences arised from the initial value of current and voltage and in minor proportion with the particular conditions of the WiFi network channel. We next present the two most different found experiments (we code the experiments as OBTx-1 and OBTx-2 from now on).

### 3.1. Power Consumption in T_c_

We above presented the charging current and voltage curves of the isolated battery lasting 6 h. But now we present the same curves for OBTx-1 and OBTx-2 when the battery was used in our Mechanism.

**Figure 8:**
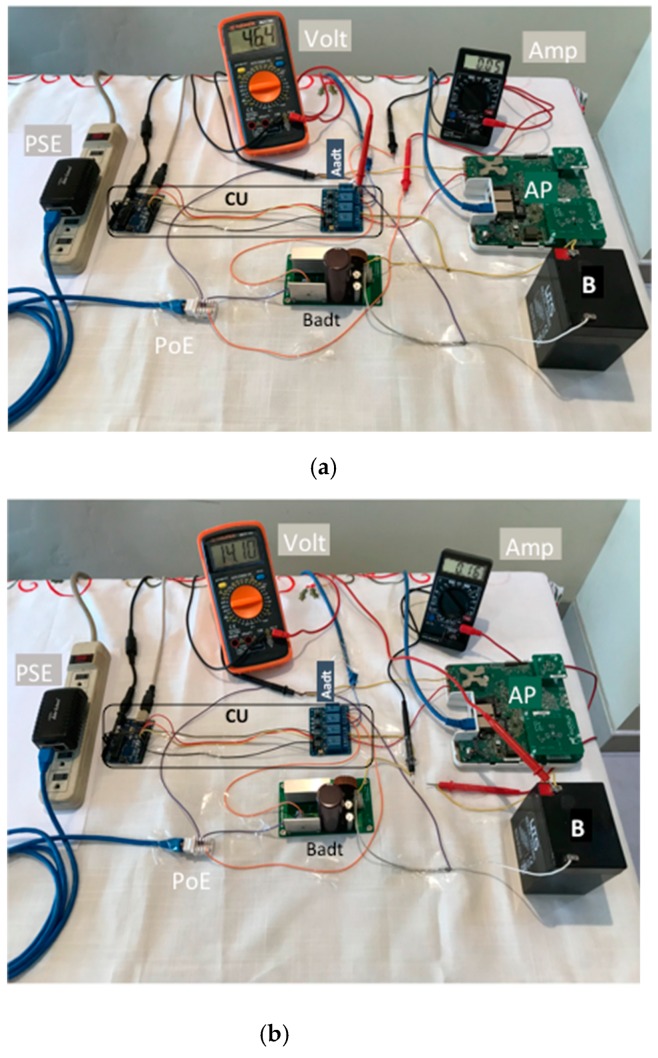
Charging current and voltage of the B with our mechanism: (**a**) the power-over-ethernet (PoE) output, (**b**) the Badt output.

Figure 8 shows a photo with the mounted hardware elements and interconnections and measurements in the volt and amp devices. The PoE was connected (orange cable) to the CU (which distributed *V* for the Aadt and Badt), the Aadt and the Badt (lilac cables). We made measurements for the:

• PoE output (Figure 8a):

1. *V* (volt): black cable tip (-) of volt was connected to the Aadt and the red cable tip of Volt (+) to the CU. Finally, the volt measured 46.4 V.
2. *I* (amp): orange cable was intercepted by amp connecting first its black cable tip (-) to the PoE output and the red cable tip (+) to the CU. Finally, the amp measured 0.05 A.

• Badt output (Figure 8b):

1. *V_c_* (volt): black cable tip (-) of volt was connected to the Aadt and the red cable tip of volt (+) to positive terminal of *B*. The *V_c_* shown in the figure corresponds to 98.6% of the total voltage charged, the volt measured 14.1 V.
2. *I_c_* (amp): black cable tip (-) of volt was connected to a yellow cable connected to the CU and the red cable tip of *Volt* (+) to a yellow cable connected to the positive terminal of *B.* The *I_c_* shown in the figure corresponded to 98.6% of the total voltage charged, the amp measured 0.16 A.

The values of *V* and *I* were enough to keep the 2.4 and 5 GHz WiFi interfaces of the Ruckus AP working and charging the lead acid battery (*B*).

**Figure 9:**
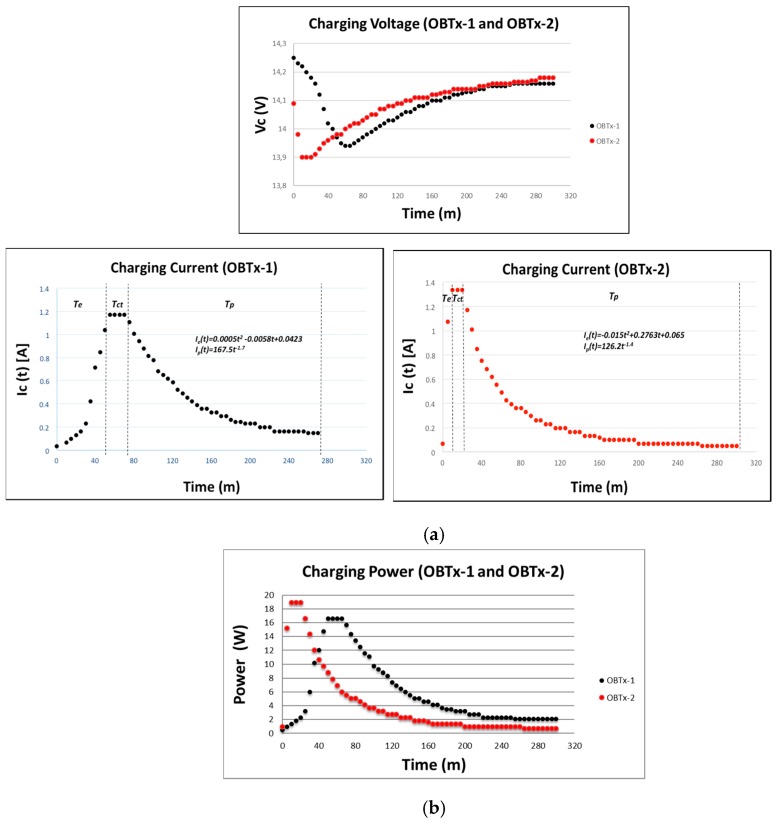
(**a**) Evolution of *V_c_* and *I_c_* in time. (**b**) Evolution of power consumption in time (*T_c_*).

As expected (from literature and our formal model) the *V_c_* and *I_c_* varied along time as shown in Figure 9. Initially the battery had a vacuum voltage (before starting Charge period) of 3 V (OBTx-1) and 8.34 V (OBTx-2). When connected to the Aadt (and PoE) the *V_c_* increased to 14.25 V (OBTx-1) and 14.09 V (OBTx-2). In both tests the *V_c_* falled to a minimum value (valley) and then it reached a maximum value close to 14.2 V which marked the value of *T_c_* around 270 m. The most significant difference occurs in the first 45 m (around 0.32 V); that difference was reduced in 210 m to 0.05 V and 0 V at 320 m. These differences were due to the vacuum voltage. We found the values of coefficient $w_{v}=3.24$. We separately represented *I_c_* because we differentiated three intervals of time (*T_e_*, *T_ct_* and *T_p_*) with vertical dashed lines. The end of *T_e_* (starting of *T_ct_*) for *OBTx-1* (55 m) was greater than OBTx-2 (10 m) due to vacuum voltage. During *T_e_* the demand of *I_c_* increased to the maximum (100% for OBTx-1 and OBTx-2) of overall process; and the *V_c_* increased to 98.11% (OBTx-1) and 95.2% (OBTx-2) and then the *V_c_* increased slowly until reach *T_c_*. Due to in *T_e_* the Aadt used 0.04 A, the Badt was able to deliver enough current to charge the battery without extra power consumption provided by the PSE. Current stabilization (*w_t_*) was achieved at 1.17 A (OBTx-1) and 1.33 A (OBTx-2) during 15 and 10 m respectively (*T_ct_*). During *T_ct_* the *V_c_* was almost constant. During *T_p_* the current approached 0.15 A (OBTx-1) and 0.05 A (OBTx-2) following a negative potential trend. We found the values of coefficient $w_{c}=3.25$. The curves of power consumption had the same shape as the curves of *I_c_* scaled by *V_c_*. Maximum consumption was reached at *T_e_* and then decayed at approximately 2 W (OBTx-1) and 0.7. W (OBTx-2). Because the battery consumed a limited amount of energy supplied by PSE.

### 3.2. Power Consumption in Td

We assumed the battery was charged (it had reached the maximum value of *I_c_* and *V_c_* defined in the previous Section) for analyzing *T_d_*. We proceeded to analyze power consumption in *T_d_* keeping the WiFi interfaces of Ruckus AP active and transmitting only beacons. Now the I_d_ flowed from the battery to the Ruckus AP through Aadt and Badt (the PR left the PSE in high impedance).

**Figure 10:**
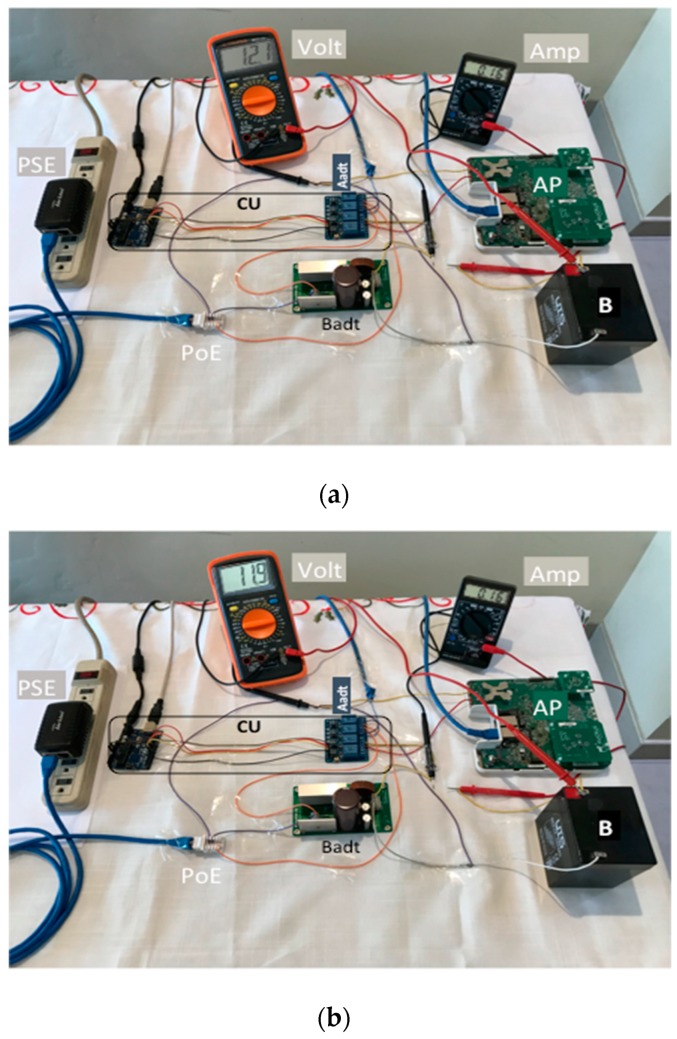
Discharging current (*I_d_*) and voltage (*V_d_*) of the battery with our mechanism: (**a**) OBTx-1 and (**b**) OBTx-2.

Figure 10 shows two photos with the volt and amp devices for measuring *I_c_* and *V_c_* for OBTx-1 and OBTx-2. We did measurements for the:

- *V_d_* (volt): black cable tip (-) of volt was connected to the *Aadt* and the red cable tip of volt (+) to positive terminal of *B*. Finally, when the *V_d_* had reached 84.5% (OBTx-1) and 88.9% (OBTx-2) the volt measured 12.1 V (OBTx-1) and 11.9 V (OBTx-2).
- *I_d_* (amp): black cable tip (-) of volt was connected to a yellow cable connected to the CU and the red cable tip of volt (+) to a yellow cable connected to the positive terminal of *B.* Finally, when the *V_d_* had reached 84.5% (OBTx-1) and 88.9% (OBTx-2) the amp measured 0.16 A (OBTx-1 and OBTx-2).

**Figure 11:**
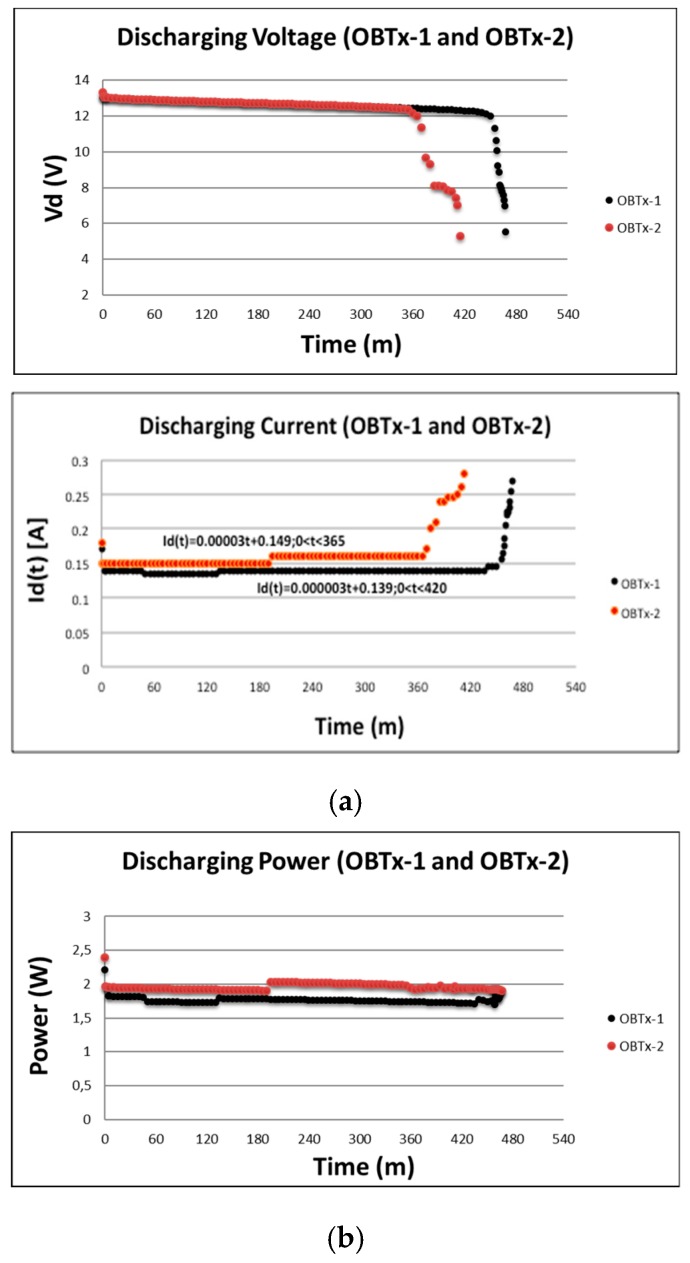
(**a**) Evolution of *V_d_* and *I_d_* in time. (**b**) Evolution of power consumption in time (*T_d_*).

As expected (from literature and our formal model) the *V_d_* and *I_d_* varied along time as shown in Figure 11. When the *V_d_* approached 12 V then it radically decreased its value to 7 V approximately. We experimentally observed that under that value the Ruckus AP did not work well. Therefore, that value marked the *T_d_*. That is, *T_d_* = 467 m (*OBTx-1*) and *T_d_* = 412 m (*OBTx-2*). Initially *I_d_* rapidly increased to almost 0.18 A and then dropped and stabilized at an almost constant value of approximately 0.16 A until *V_d_* dropped below the 12 V. Finally, it radically increased drastically until *V_d_* approached to 7 V. Reinforcing the value of *T_d_* observed for *V_d_*. Power consumption was in the range of 1.5 W (OBTx-1) and 2.2 W (OBTx-2) which was a low value. In both tests $w_{s}\to 0$ and $w_{f}>0$. This means *I_d_* could start from a large enough value to keep the AP working within its correct margins. This implies the need to provide a corresponding *V_d_*.

## 4. Discussion

In previous sections we explained the power consumption for *T_c_* and *T_d_* in order to show the complexity to calibrate *w* parameters of our model. In this Section we analyze the power consumption in WiFi AP (using our mechanism or not) in one cycle (*k* = 1) taking into account the power consumed in *T_c_* and *T_d_*. Then we project our results to infer the energy saving for greater values of *k*.

**Figure 12:**
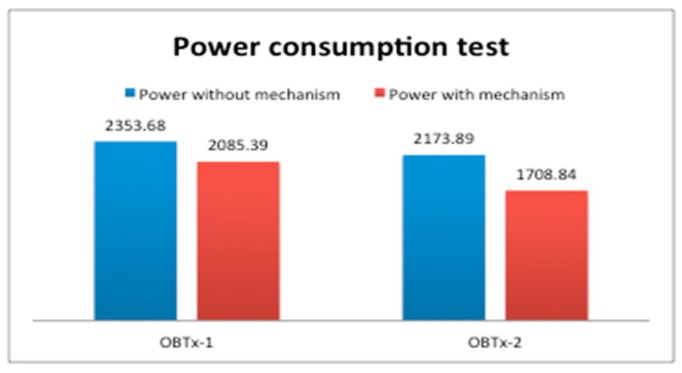
Comparison of power consumption in one cycle (*k* = 1) with and without our mechanism.

We always obtained (in all the implemented test) that $T_{d}>T_{c}$. In test OBTx-1 we obtained a difference of 3 h (7 h 30 m > 4 h 30 m), and in test OBTx-2 we obtained 1 h 8 m (6 h 8 m > 5 h 0 m). Moreover, we showed in Section 2.1 that if $w_{v}>w_{c}$ then $T_{d}>T_{c}$, and in that case energy saving could be achieved. In Section 3 we showed $w_{c}=3.25$ and $w_{v}=3.24$ and $T_{d}>T_{c}$ for both tests. So, according to our formal model, energy saving in one cycle (*k* = 1) was achieved. Let us now show a power consumption analysis, in one cycle (*k* = 1), using and not using our mechanism. Figure 12 shows that in a period of 12 h (OBTx-1) or 11 h (OBTx-2), we obtained a difference of 268 W and 465 W. Althougth, not a high amount of energy was saved, our model was satisfied.

Analyzing the charging process of isolated battery versus the charging and discharging cycles of the battery using our mechanism we obtained several learn lessons:

- The vacuum voltage should be as high as posible. This allows to augment the slope of *I_c_* during *T_e_*. Which means that a constant *I_c_* will be obtained earliest. In this way we enfoster the shortening of *T_c_*.
- Starting the discharge period, the *V_d_* must be as high as possible and the slope of Id be as small as possible. This enfoster to extend *T_d_*.
- In both previous cases, if the WiFi AP works very little, we enfoster the shortening of *T_c_* and extension of *T_d_*.

Let us reason about the above results in a day of a typical official organism like a government building (*k* = 2). Usually, it has normal activity from 7 h to 17 h in a day (10 h approximately). During that time the WiFi APs can be active transmitting data. But in the rest of the day (from 17 h 01 m to 6 h 59 m) the WiFi APs will be inactive only transmitting beacons (14 h approximately). Thus, we have a margin of hours in a day to make work our Mechanism for energy saving. That is, in that period of time of 14 h, around 6 h the WiFi AP will not consume energy of the PSE. This is because the battery is charged with no extra power consumption from PSE.

**Figure 13:**
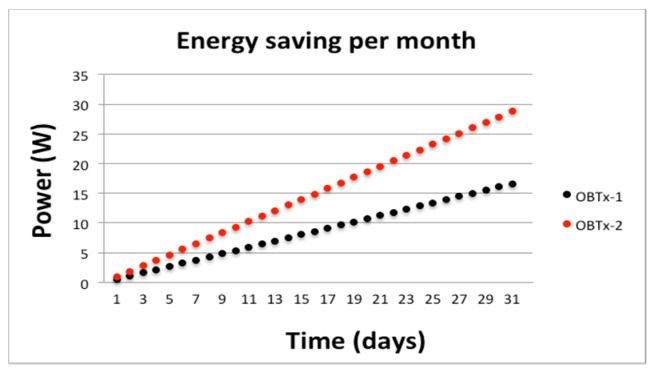
Scaling energy saving of a day (*k* = 2) in a month (*k* = 62).

For greater values of *k* we can use a simulation based on WoS starting from our experimental values of *T_c_* and *T_d_*. That analysis will inform us about the probability to save an estimated amount of energy (W). We think it is more interesting to scale the energy saving of two cycles (*k* = 2) per day, for example in one month (directly expanding it to a bigger amount of time). Figure 13 shows the scaled energy saving in a month that will reach 28.8 KW, which is very convenient because it could save 345.6 KW annually for each AP in operation. In a typical medium-size government building, with around 50 WiFi AP, the energy saving would be considerable.

A final remark about the optimization of energy saving derived from our formal model is made. This remark has been exposed also in the experimental results previously presented. The *T_c_* can be minimized if the *I_c_* will be minimized and vacuum voltage of the battery will be maximized when adjusting properly the implied parameters (*w*). The *T_d_* can be maximized if the battery voltage remains greater than a voltage threshold.

## 5. Conclusions

In this paper we presented a hardware mechanism for energy saving in wireless fidelity APs. The Mechanism can be used with any AP and any voltage supply. We designed a parametric formal model for maximizing the energy saving. We implemented in hardware our novel mechanism and we verified the formal parametric model with the experimental results. It was shown that when the AP only transmits signaling wireless fidelity beacons, energy saving can be obtained. Projection of the amount of saved energy in a year is important in case it would be applied to a medium-high amount of APs.

The process to find an appropriated battery for implementing the hardware mechanism was not a simple task. Moreover, finding standard voltage and current adapters was also hard task. One lesson learned is that the conditions under the battery can be used for energy saving assuring a vacuum voltage and minimizing the current it consume. Another lesson is the need to maintain the battery working under certain intervals of voltage and current load in order to assure the maximum energy saving.

Interesting experimental results aim us to define future work along several lines. We highlight four of them:

- To add clean and free energy supply to the system in order to help charging the battery in conjunction with the standard energy supply like power over ethernet (used in this paper) or another, modification of the adapters and power router will be needed.
- To study the influence of the activity of the WiFi AP transmitting user data, we did some initial tests considering low constant bit rate traffic in the WiFi AP and obtained also energy saving. Those tests must be reinforced in order to infer good conclusions. Moreover, it is difficult to design a formal model that could explain the realistic conditions for obtaining energy saving. In addition, if the AP is active (transmitting user data) most of the time, careful study will be necessary to analyze the possible packet loss during the transition between the discharge and charge cycles.
- To determine the influence of the wireless fidelity channel in energy saving. In dense wireless fidelity networks there are a lot of interferences among APs, and the number of wireless fidelity terminals is very high. Usually the APs change of channel producing more energy consumption. Formalizing realistic conditions for energy saving is very hard and the changing of our power routing will be also needed.
- The constant charging and discharging of the battery over a long amount of time (2 or more years) causes its capacity to be reduced considerably or even makes it cease working. This could complicate the charge period being shorter than the discharge period for achieving energy saving (after that long amount of time). Properly selecting and planning a battery replacement to minimize this effect is a key factor. On the other hand, the energy-saving model must be extended to introduce this correctly.
